# How environment and genetic architecture of unreduced gametes shape the establishment of autopolyploids

**DOI:** 10.1038/s41437-025-00816-3

**Published:** 2026-01-13

**Authors:** Yu Cheng, Filip Kolář, Roswitha Schmickl, Josselin Clo

**Affiliations:** 1 Department of Botany, Faculty of Science, Charles University, Prague, Czech Republic; 2 Institute of Botany, Czech Academy of Sciences, Průhonice, Czech Republic; 3 CNRS, Univ. Lille, UMR 8198–Evo-Eco-Paleo, Lille, France

**Keywords:** Evolutionary theory, Polyploidy in plants

## Abstract

It is broadly assumed that polyploidy success results from increased fitness associated with whole genome duplication due to higher tolerance to stressful conditions. In agreement, several theoretical models found that, among other factors, a better tolerance to new environmental conditions can promote polyploidy establishment. Here, we investigated the effect of the genetic and environmental factors affecting the architecture of unreduced gamete production, to see how it affects the origin and persistence of autopolyploids in both stable and disturbed environments. We developed a theoretical model in which we modeled the joint evolution of a quantitative trait under selection and the production of unreduced gametes; both traits were pleiotropically linked. We followed the adaptation of initially diploid populations to a new environment to which tetraploid individuals were directly adapted. The generation of these autotetraploid individuals was enabled by the genetic production of unreduced gametes and by the environmental change modifying the average production of these gametes. We found that for realistic values of unreduced gamete production adaptation to new environmental conditions was mainly achieved through adaptation of diploids to the new optimum rather than the persistence of newly adapted tetraploid individuals. In broader parameter sets, we found that the adaptation process led to mixed-ploidy populations, except when the populations were swamped with unreduced gametes, and that pleiotropy and environmental effects favored the co-existence of both cytotypes.

## Introduction

Polyploids are organisms known for having more than two chromosome sets compared with their diploid progenitors. Polyploidy, or whole genome duplication (WGD), is frequently accompanied by meiotic abnormalities, and it alters gene dosage, consequently affecting the phenotypes and long-term evolution of populations (Bomblies 2023; Doyle & Coate 2019; Otto 2007). There is evidence that polyploidization is an important mechanism in speciation and diversification (Soltis et al. 2015), with blooms of diversity associated with ancient WGD events, both in plants, particularly the angiosperms, animals, and fungi (Albertin & Marullo 2012; Gregory & Mable 2005; Jiao et al. 2011; Van De Peer et al. 2023). For example, it has been suggested that all angiosperms descended from an ancient WGD event (Jiao et al. 2011), and 25 to 35% of angiosperm species have speciated through WGD (Mayrose et al. 2011; Wood et al. 2009). The geographic distribution of polyploids in the angiosperms shows a latitudinal trend, with a higher frequency of polyploids at higher latitudes (Rice et al. 2019). It is an ongoing debate if polyploidy fosters adaptability to stressful environments (Madlung 2013), and if in particular recently formed polyploids are associated with stressful conditions, such as cold and dry environments, as reviewed by Van De Peer et al. (2021). This potential environmental association becomes particularly intriguing when considering the mechanisms leading to the formation of polyploids.

Although somatic doubling could potentially play a role in the formation of polyploids (Bachmann et al. 2021), it is currently assumed that it usually involves unreduced gametes (UG), which are gametes with the somatic ploidy level, such as diploid gametes in a diploid organism; for the cytological mechanisms and molecular regulatory networks underlying UG formation see Brownfield & Kohler (2011) and De Storme & Geelen (2013). Pathways to polyploidy can be (i) ‘one-step’, by the fusion of two unreduced gametes; rather rare in intraspecific crosses because of the rarity of UG formation, but rather common in interspecific crosses, as UG formation is higher in hybrids, or (ii) via the ‘triploid bridge’, by the fusion of unreduced and reduced gametes within a diploid population, with triploid offspring producing unreduced gametes that backcross with the reduced gamete parent (Mason & Pires 2015). UG formation has been shown to depend on abiotic and biotic factors, such as temperature, moisture, nutrition and herbivory (Kreiner et al. 2017a; Mason & Pires 2015). Polyploids can be classified into two categories, autopolyploid and allopolyploid, based on their taxonomic origin or homolog pairing preferences and mode of inheritance (Bomblies, 2023; Lv et al., 2024). Autopolyploids have chromosome sets from a single species and undergo polysomic inheritance. Allopolyploids have chromosome sets from two different species and undergo disomic inheritance.

Once polyploids are formed, they face numerous challenges to finally establish in a population and to form a distinct polyploid lineage that may eventually speciate from its diploid progenitor. Upon emergence, polyploids tend to experience a lack of mating opportunities (minority cytotype exclusion; Levin 1975). The lack of mating opportunities in short-lived polyploid organisms can be theoretically overcome by a strong fitness advantage of polyploids (Husband, 2004, but see Porturas et al., 2019; Clo and Kolář, 2021 for contradiciting empirical data), polyploidy-associated mating system transitions (Griswold 2021; Oswald & Nuismer 2011), higher clonal reproduction of polyploids (Van Drunen & Friedman 2022), but also certain spatial components of polyploid establishment (Kauai et al. 2023; Spoelhof et al. 2020). All these components of polyploid establishment received considerable attention from a theoretical point of view (see for example Felber 1991; Husband 2004; Gaynor et al. 2023; Griswold 2021; Kauai et al. 2023; Oswald & Nuismer 2011; Van Drunen & Friedman 2022), allowing us to better understand how these major parameters and their interactions allow (or not) the maintenance, and sometimes fixation, of polyploid individuals in initially diploid populations or in new environments.

More recently, the role and evolution of UG production in polyploidy evolution have received new attention. UG production per se is assumed to come with an immediate fitness disadvantage due to meiotic mishap. In the formation of unreduced gametes, several genes can be involved (Bretagnolle & Thompson 1995; Brownfield & Kohler 2011; De Storme & Geelen 2013), for which pleiotropic effects (i.e. the fact that several traits are affected by a single locus) with male and/or female fitness have been found (Brownfield & Kohler 2011), with mutations increasing UG production having generally a negative effect on plant fitness (Brownfield & Kohler 2011). For example, the *dyad* allele of the *SWITCH1 (SWI1)/DYAD* gene, leading to defects in female meiotic prophase I and female UG production, also affects the final number of viable ovules, with plants only producing 1-10 viable seeds (Brownfield & Kohler 2011).

Historically, the genetic architecture of UG production is omitted in models, and UG production is considered a fixed quantity that can vary among parameter sets of simulation models. It is known from empirical data that in natural populations of extant angiosperm populations the UG production is generally low in non-hybrid species (between 0.1 to 2%; Kreiner et al., 2017b). Few attempts were made to model in which conditions such low rates of UG formation can be found, and to model the effect of the evolution of UG production in time on the probability of polyploid fixation (Clo et al. 2022; Gerstner et al. 2024). Clo et al. (2022) investigated the evolution of UG as a quantitative trait and identified that when genetic drift is strong, there will be increased UG formation; therefore, autopolyploidy may fixate in the population. Gerstner et al. (2024) modeled the inter-generation variation of UG following the empirical data by Kreiner et al. (2017b), and found that such variation can lead to the persistence of both autopolyploids and their diploid progenitors. While both models are informative, they are built on strong assumptions. Clo et al. (2022) made the genetic architecture of UG production too simplistic, omitting, for example, the fact that mutations increasing UG production are pleiotropically linked to fitness and strongly affect fitness components such as pollen and/or ovule production (d’Erfurth et al. 2010; Erilova et al. 2009; Ravi et al. 2008; Wang et al. 2010; see also Brownfield & Kohler 2011 for a review). Clo et al. (2022) also neglected the effects of the environment, while factors like temperature are known to have major effects on UG production rates (De Storme et al. 2012; Mason et al. 2011; Pecrix et al. 2011; Schindfessel et al. 2023; Wang et al. 2024). Since UG production is determined by both environmental and genetic factors (Parrott & Smith 1986; Tavoletti et al. 1991a), considering a more realistic genetic architecture of UG production could strongly impact the conclusions of the above-mentioned models, and could make us understand the relative contribution of both genetic and environmental effects to UG production and polyploidy evolution.

We investigated the effect of genetic and environmental factors affecting UG production, to see how it influences the origin and establishment of autopolyploids in both stable and disturbed environments. We built an individual-based simulation model, in which we tested how a complex genetic architecture of UG production affects the origin and potential maintenance of polyploid lineages. In this model, pleiotropy with the fitness of individuals is introduced, coupled with an effect of the environment on the average level of UG production. In conclusion, we found that for realistic values of UG production adaptation to new environmental conditions was mainly realized by adaptation of diploids to the new optimum rather than the fixation of newly adapted tetraploid individuals. In broader parameter sets, we found that the adaptation process led to mixed-ploidy populations, except when the populations were swamped with unreduced gametes, and that pleiotropy and environmental effects favored the co-existence of both cytotypes.

## Material and methods

### General description of the model

The model is built as follows. We considered a Wright-Fisher model of a quantitative trait under selection. We considered non-overlapping generations, a fixed population size (*N* = 200 individuals), and obligately outcrossing individuals. After selection, parents produce gametes. We considered both reduced and unreduced gametes. In our model, the merging of unreduced gametes of two diploid individuals is the only path to autopolyploidy, and only diploid and tetraploid cytotypes are considered. The model works in two steps: a first step in which the population is under stabilizing selection up to reaching a mutation-selection-drift (MSD) equilibrium, followed by a second step in which an environmental change is introduced by changing the phenotypic optimum and by adding an effect of the new environment on the average UG production in the population. We considered that tetraploid individuals are immediately adapted to the new conditions, but not diploids. The step of stabilizing selection stops when the population is at M-S-D equilibrium, that is, when the average population fitness value calculated over the last thousand generations does not differ by more than one percent from the mean fitness calculated over the previous thousand generations. The step of directional selection lasts 500 generations. More details are given below.

### Life cycle of individuals

**Fig. 1:**
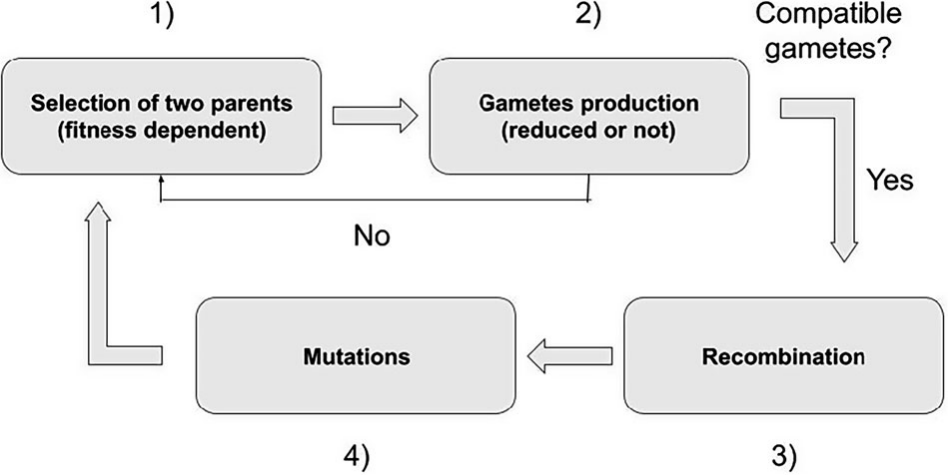
Schematic representation of the life cycle of individuals. First, parents are selected based on a weighted sample. Once two different parents are sampled, the kind of gametes they produce is defined. If the merging of gametes leads to a diploid or a tetraploid offspring, the life cycle continues; if not, two new reproducers are sampled. Once the kind of gametes produced has been determined, recombination and mutations (underlying the phenotypic trait and the trait coding for unreduced gamete production) are introduced. The reproduction phase ends when *N* = 200 new offspring have been generated.

The life cycle can be summarized in four successive events. First (1), there is a fitness-dependent choice of the two parents (selection). As we assumed that populations are obligatorily outcrossing, the two parents need to be different. The number of offspring an individual can contribute to the next generation is not limited. Selection takes place as follows: an individual is sampled randomly among the *N* individuals, but its sampling probability is weighted by its fitness. Once the two parents are chosen, (2) the type of gamete (reduced or unreduced) they will produce is chosen. For each reproducer, one number is sampled in a uniform distribution between 0 and 1. If the rate of UG production (*p*_UG_, male or female) is higher than the sampled value, the reproducer generates an unreduced gamete. For reduced (*n*) gametes (*n* = x and *n* = 2x gametes for diploid and tetraploid individuals, respectively), one allele per locus of each sister chromatid is sampled randomly for each trait. This phase is then followed by (3) the introduction of mutations for each trait, the number of which is sampled from a Poisson distribution with parameter *U*, the haploid genomic mutation rate (with *U* = *µL*, where *µ* is the per-locus mutation rate and *L* is the number of loci underlying the trait under study). Finally (4), the gametes are merged to form the offspring. If the resulting offspring is not diploid or tetraploid, it will not contribute to the next generation. The reproduction phase stops when *N* viable offspring are formed. The life cycle is summarized in Fig. 1.

### Genetic architecture of traits

We considered the joint evolution of a quantitative trait *Z* and the production of unreduced gametes (UG); their genetic architectures are the following:

1. Genetic architecture of the phenotypic trait under selection:
  The phenotypic value *z* of an individual was determined by the additive action of *L*_Z_ loci, each with an infinite possible number of alleles, and was given by
  1 $$z={g}_{z}+{e}_{z},$$
  where *g*_z_ was the genetic component of the individual’s phenotype and was given by *g*_z-2x_ = ${\sum }_{j}^{L}({g}_{j}^{M}+{g}_{j}^{P})$, where *g*^M^_*j*_ (respectively *g*^P^_*j*_) was the additive allelic effect at locus *j* inherited from the maternal (respectively paternal) gamete in the diploid population. After polyploidization and with tetrasomic inheritance, the genetic component became g_z-4x_ = ${\sum }_{j}^{L}d.({g}_{j}^{M1}+{g}_{j}^{M2}+{g}_{j}^{P1}+{g}_{j}^{P2})$, where *g*^M1^_*j*_ and *g*^M2^_*j*_ (respectively *g*^P1^_*j*_ and *g*^P2^_*j*_) were the additive allelic effects at locus *j* inherited from the maternal (respectively paternal) gametes. The parameter *d* controlled for the dosage effect and determined the effect of polyploidization on the tetraploid genotypic values compared to diploid ones. The additive value of a new mutant allele was drawn from a Gaussian distribution of mean 0 and variance *a²*. The random environmental effect on the phenotypic trait *e*_z_ was drawn from a Gaussian distribution of mean 0 and variance *V*_E_ and was considered to be independent of the genetic components of fitness. The effect of the environment on the phenotypic trait under selection is fixed during all steps of the simulation.
  The trait underwent stabilizing selection around an arbitrary optimal phenotypic value, denoted *Z*_opt_ and being equal to zero in this model. The fitness value *W*_Z_ of an individual with phenotype *z* was thus described by the Gaussian function:
  2 $${W}_{z}={e}^{-{\delta }^{2}/2{\omega }^{2}},$$
  where *δ* was the distance between the individual’s phenotype *z* and the optimum trait value and *ω*² was the width of the fitness function, representing the strength of selection. There was no dominance at the phenotypic scale in this model, but recessivity of mutations arose naturally at the fitness scale due to the non-linearity of the phenotype-fitness function (see Manna et al. 2011 for diploids and Clo, 2020 for tetraploids).
2. Genetic architecture of UG production

The trait “unreduced gamete production” was coded for both male and female reproductive functions separately (as supported in Kreiner et al., 2017a), by *L*_G-M_ = *L*_G-F_ freely recombining bi-allelic loci. The average UG production for male and female functions *p*_UG-M_ and *p*_UG-F_ of an individual was determined by the additive action of alleles at different loci, each with an infinite possible number of alleles, and was given by

3.1 $${p}_{{\rm{UG}}-{\rm{M}}}={g}_{{\rm{UG}}-{\rm{M}}}+{e}_{{\rm{UG}}}$$

and

3.2 $${p}_{{\rm{UG}}-{\rm{F}}}={g}_{{\rm{UG}}-{\rm{F}}}+{e}_{{\rm{UG}}},$$

where *g*_UG-M_ (respectively *g*_UG-F_) was the genetic component of the individual’s male (respectively female) UG production and was given by *g*_UG-M-2x_ = ${\sum }_{j}^{L}({g}_{j}^{{UG}-M-M}+{g}_{j}^{{UG}-M-P})$(respectively *g*_UG-F-2x_ = ${\sum }_{j}^{L}({g}_{j}^{{UG}-F-M}+{g}_{j}^{{UG}-F-P})$), where *g*^M^_*j*_ (respectively *g*^P^_*j*_) was the additive allelic effect at locus *j* inherited from the maternal (respectively paternal) gamete in the diploid population. After polyploidization and with tetrasomic inheritance, the genetic component became *g*_UG-M-4x_ = ${\sum }_{j}^{L}({g}_{j}^{{UG}-M-M1}+{g}_{j}^{{UG}-M-M2}+{g}_{j}^{{UG}-M-P1}+{g}_{j}^{{UG}-M-P2})$, where *g*^M1^_*j*_ and *g*^M2^_*j*_ (respectively *g*^P1^_*j*_ and *g*^P2^_*j*_) were the additive allelic effects at locus *j* inherited from the maternal (respectively paternal) gametes. The additive value of a new mutant allele was drawn from a Gaussian distribution of mean 0 and variance *a²*. The environmental effect *e*_UG_ was fixed during each step of the simulations but could vary between the two steps of the simulation process. During the first step (stabilizing selection), *e*_UG_ equals zero. During the second step (directional selection), *e*_UG_ can be equal to 0, 0.1 or 0.3 (Table 1). If *p*_UG-M_ and/or *p*_UG-F_ were < 0 (respectively > 1), then *p*_UG-M_ and/or *p*_UG-F_ were forced to be equal to 0 (respectively 1). In this model, we simulated three scenarios of pleiotropy: (1) all the different loci of the different traits can be independent, (2) pleiotropy between loci involved in UG production for male and female functions can happen (in such a case we used the female genetic basis as a reference for both sexes for the pleiotropic loci under study), or (3) pleiotropy between all kinds of loci (phenotypic trait and unreduced gametes for male and female functions) can occur. These different scenarios fit with what is known in the model plant *A. thaliana* (Brownfield & Kohler 2011).

**Table 1:** Description of the model parameters, their abbreviations, and tested values in simulations.

Parameter	Abbreviation	Value(s)
Population size	*N*	200
Number of loci – Quantitative trait	*L*	100
Number of loci – UG production	*L*_G-M_ - *L*_G-F_	100
Number of pleiotropic loci	*-*	0, 40 or 80
Number of replications	*-*	100
Pleiotropy scenario	*-*	No, among female and male UG loci or among all traits
Haploid genomic mutation rate	*U*	0.005, 0.05 or 0.1
Variance of mutational effects	*a²*	0.05
Dosage effect	*d*	0.65
Variance of environmental effects	*V*_E_	1
Strength of stabilizing selection	*ω²*	9
Environmental effect on UG production	*e*_UG_	0, 0.1 or 0.3

### Simulations and parameter sets

Simulations were run for 81 parameter sets (3 mutation rates, 3 pleiotropy scenarios, 3 different numbers of pleiotropic loci, 3 different strengths of the environment on UG production). Only the most representative sets are described in the manuscript. For each parameter set, 100 simulations were performed to generate the mean and variance of the metrics of interest. The values chosen for the mutation rate *U* were 0.005, 0.05 and 0.1, reflecting the per-trait haploid genomic mutation rate found in the literature (Halligan & Keightley 2009). We used parameter set values similar to those explored in similar theoretical models allowing comparisons among studies, with the number of freely recombining loci under selection *L* = 100 (as in Clo et al. 2022, but little empirical knowledge exists and therefore the number of loci could be much smaller), a² = 0.05 (Bürger et al. 1989; Ronce et al. 2009), and var(e_Z_) = var(e_UG_) = 1 (Bürger et al. 1989; Oswald & Nuismer 2011; Ronce et al. 2009). The strength of stabilizing selection was set to *ω*² = 9 to fit empirical observations (Clo & Opedal 2021). We considered a population size of *N* = 200 individuals. The dosage parameter *d* was equal to 0.65 (*g*_Z-4x_ = 1.3*g_Z-2x_, which is found in synthetic neo-polyploid and established polyploid populations on average; see Porturas et al., 2019 and Clo & Kolář, 2021 for meta-analyses). When pleiotropy was considered, the number of pleiotropic loci was set equal to 0 (no pleiotropy), 40 (moderate pleiotropy), or 80 (high pleiotropy), which fits the range found in the genomic literature (McKay & Anholt, 2024), although nothing is known about the number of pleiotropic loci affecting UG production. As mentioned earlier, before the environmental change the fitness of polyploids was close to that of diploids (slightly lower because of the gigas phenotypic effect), but was initially higher at the beginning of the environmental change. Before the change, e_UG_ equaled 0 but could be equal to 0, 0.1 or 0.3 after the change, when considering that the environmental stress (such as change in temperature) also affects UG production. Finally, *Z*_opt_ switched from 0 to 2.5 after the environmental change. A summary of the parameters used and their values can be found in Table 1.

### Metrics of interest

The metrics measured within populations were: frequency and genetic variance (note that the phenotype variance followed similar variation in the simulations; Fig. S1) of unreduced gametes, average fitness of the population, and frequency of tetraploids in the population at each generation. It is reasonable to infer the mean and variance of genetic UG production by taking the average value of male and female functions because both metrics are similar in simulations (see Fig. S2 for an example). This was due to the small variations in reproductive success and UG production among individuals, because of the null average mutational effects and their small variance. The mean and variance of the above-mentioned metrics are inferred from 100 independent replicates per parameter set.

## Results

### Description of populations at mutation-selection-drift equilibrium

**Fig. 2:**
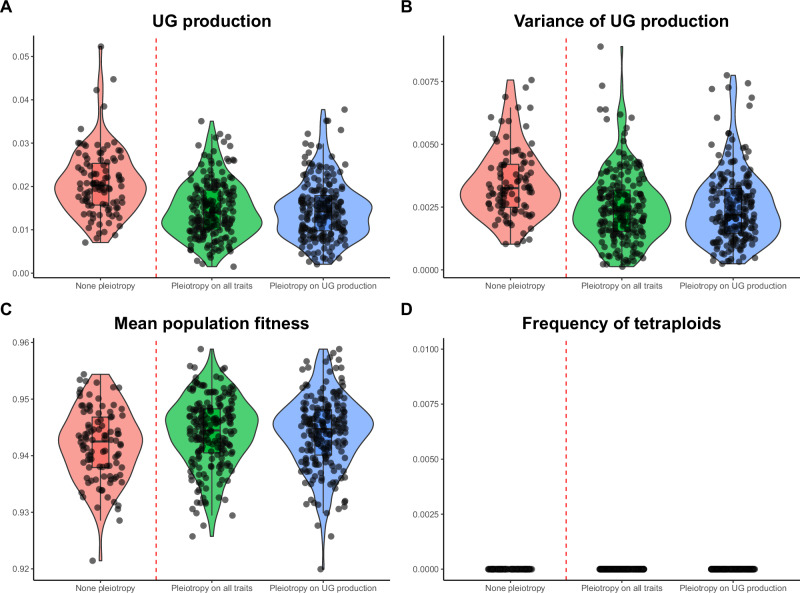
Metrics of interest for populations at M-S-D equilibrium. Effect of pleiotropy scenario (none, all traits, unreduced gametes; number of pleiotropic loci = 80) on unreduced gamete production and its genetic variance, population fitness, and tetraploid frequency at the mutation-selection-drift equilibrium, simulated from a population of genetically identical diploid individuals under the mutation rate *U* = 0.005. Simulations were run for 100 replicates; black dots represent individual replicates. Pleiotropy on all traits means that pleiotropy affected both the quantitative trait under directional selection and unreduced gamete production. **A** Proportion of unreduced gamete production in diploid individuals; **B** variance of unreduced gamete production in diploid individuals; **C** population fitness; **D** frequency of tetraploids in the population.

Under stable environmental conditions (i.e. *Z*_opt_ = 0), the main factors affecting population metrics were pleiotropy and the mutation rate (Fig. 2; Supplementary Figures 3 and 5). For a given mutation rate (*U* = 0.005; see below for details), pleiotropy on the quantitative trait and on UG production generally led to more efficient purging of deleterious mutations on both traits. This resulted in lower UG production (median 1% with pleiotropy vs. median 2% without pleiotropy; Fig. 2A), lower genetic diversity for UG production (median 0.18% with pleiotropy vs. median 0.30% without pleiotropy; Fig. 2B), and slightly increased population fitness (median 94.4% with pleiotropy vs. median 94.2% without pleiotropy; Fig. 2C). The genetic variance for UG production was small (Fig. 2B), meaning that the UG production is relatively stable among individuals. Higher mutation rates (*U* = 0.05 and 0.1) did not change the patterns qualitatively, but generally led to higher average genetic UG production (median 18% without pleiotropy for *U* = 0.05 vs. median 35% for *U* = 0.1; Supplementary Figs. 3 and 4), higher genetic variance of unreduced gametes (median 18% without pleiotropy for *U* = 0.05 vs. median 36% for *U* = 0.1; Supplementary Figs. 3 and 4), and lower population fitness (median 91% without pleiotropy for *U* = 0.05 vs. median 85% for *U* = 0.1; Supplementary Figs. 3 and 4). Under low mutation rates, we did not find mixed-ploidy populations at the M-S-D equilibrium (0% of tetraploids, the populations were purely diploid; Fig. 2D), while intermediate and high mutation rates led to mixed-ploidy populations (between 1 to 10% of tetraploids, the higher when no pleiotropic effects were modeled; Supplementary Figs. 3 and 4). Because the simulations with low mutation rates gave the most realistic pattern of genetic UG production with respect to empirical rates of UG production (1–2%), we describe separately the evolution of polyploidy with this biologically realistic scenario in the first time and other scenarios in the second time.

### The evolution of polyploidy under environmental change for realistic parameter sets

**Fig. 3:**
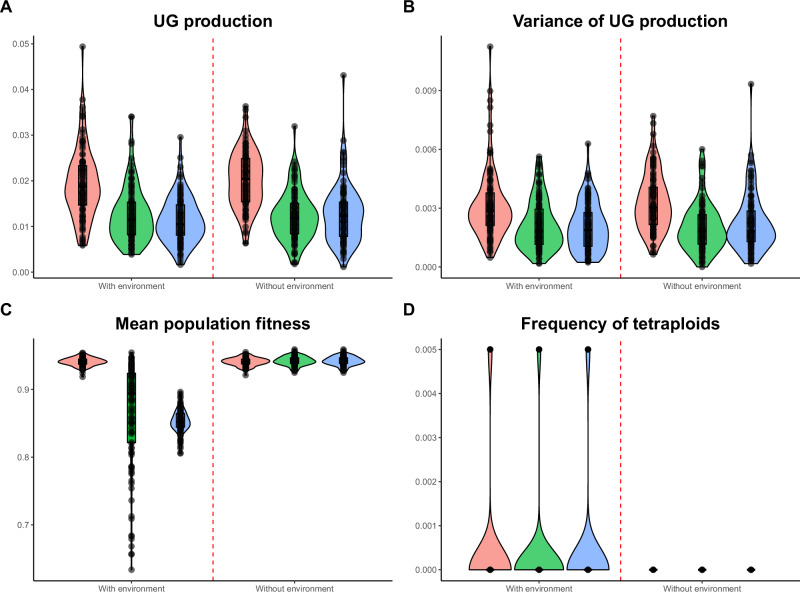
Metrics of interest after the environmental change under realistic simulation sets. Effect of pleiotropy scenario (none, all traits, unreduced gametes; number of pleiotropic loci = 80) and environmental factor (0.1) on the evolution of tetraploidy after 500 generations of directional selection, under the mutation rate *U* = 0.005. Simulations were run for 100 replicates, each spanning 500 generations; black dots represent individual replicates. Pleiotropy on all traits means that pleiotropy affected both the quantitative trait under directional selection and unreduced gamete production. **A** Proportion of unreduced gamete production in diploid individuals; **B** variance of unreduced gamete production in diploid individuals; **C** population fitness; **D** frequency of tetraploids in the population.

**Fig. 4:**
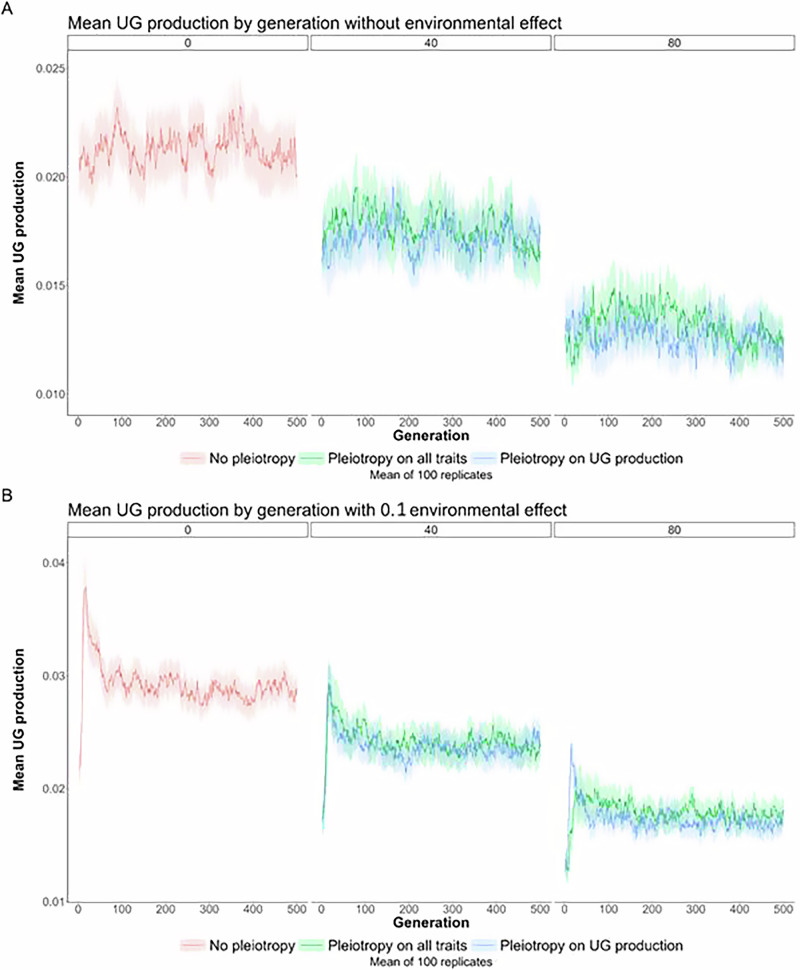
Variation of unreduced gametes production after the environmental change. Effect of pleiotropy scenario (none, all traits, unreduced gametes; number of pleiotropic loci = 0, 40, 80) on unreduced gamete production **A** without effect of environmental factor and **B** with effect of environmental factor (0.1), simulated from a population of genetically identical diploid individuals under the mutation rate *U* = 0.005 for 500 generations. Pleiotropy on all traits means that pleiotropy affected both the quantitative trait under directional selection and unreduced gamete production.

Here, we focused on simulations with low mutation rates (*U* = 0.005) and realistic environmental conditions (directional selection, *Z*_opt_ shifting from 0 to 2.5, and an environmental effect (*e*_UG_) on UG production of 10%). It was observed that environmental conditions did not significantly alter the population cytotype structure and fitness after the directional selection phase (Fig. 3). Both UG production and variance remained similar (UG production: median 2%, no pleiotropy; Fig. 3A; UG variance: median 0.3%, no pleiotropy; Fig. 3B). Mean population fitness also remained comparable after 500 generations when pleiotropy was absent (median 94%; Fig. 3C), but with both environmental effect and pleiotropy, population fitness decreased substantially, the highest being when pleiotropy only affected genetic UG production (median 85%; Fig. 3C). At the end of the simulations, the populations were only made of diploid individuals (Fig. 3D), suggesting that the recovery of fitness is made by diploid individuals adapting to new conditions rather than the invasion of newly adapted tetraploids. Environmental effects on UG production rarely led to mixed-ploidy populations (Fig. 3D). We attributed this to a conflict between environmental effect and new mutations that could have favored the selection of newly adapted tetraploids (but that had a negative pleiotropic effect on the trait under directional selection) to the detriment of the evolution of diploid individuals to the new optimum (Fig. 4B; see Fig. 4A for a comparison without environmental effect). Indeed, the genetic UG production slightly increased at the beginning of the environmental change (generally doubling in the first 20 generations, shifting for example from 2 to 4% when no pleiotropy was modeled; Fig. 4B). The production then slowly decreased during the 500 generations of adaptation (Fig. 4B).

#### The general effects of environment and genetic architecture on the evolution of polyploidy

**Fig. 5:**
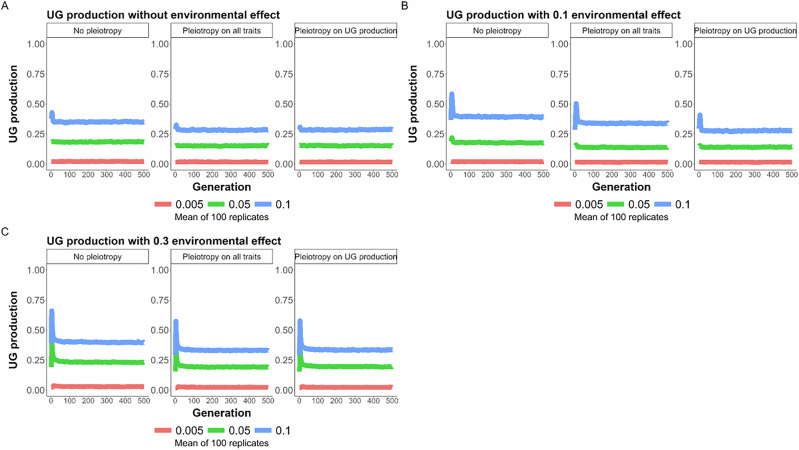
General effects of pleiotropy and environmental change on UG variation. Effect of pleiotropy scenario (none, all traits, unreduced gametes; number of pleiotropic loci = 40) and environmental factor (0, 0.1, 0.3) on unreduced gamete production under different mutation rates (*U* = 0.005, 0.05, 0.1; differently colored lines) for 500 generations. The line represents the mean result of 100 replicated simulations, each spanning 500 generations. Pleiotropy on all traits means that pleiotropy affected both the quantitative trait under directional selection and unreduced gamete production. **A** No environmental factor; **B** environmental factor 0.1; **C** environmental factor 0.3.

**Fig. 6:**
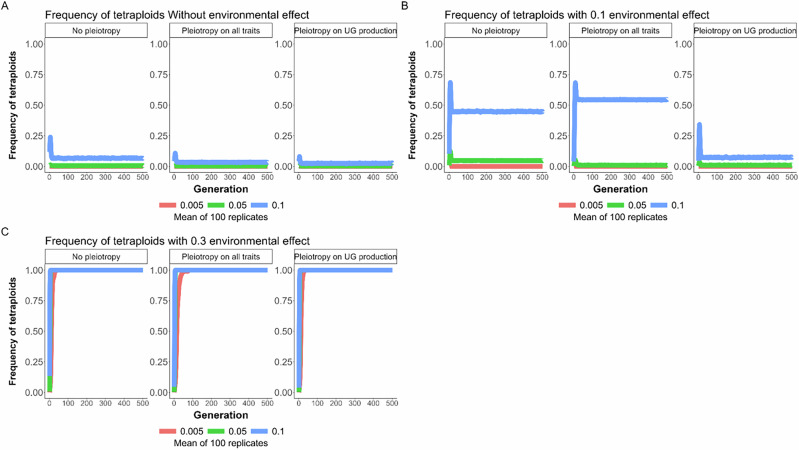
General effects of pleiotropy and environmental change on the evolution of polyploid individuals. Effect of pleiotropy scenario (none, all traits, unreduced gametes; number of pleiotropic loci = 40) and environmental factor (0, 0.1, 0.3) on the frequency of tetraploids in the population under different mutation rates (*U* = 0.005, 0.05, 0.1; differently colored lines) for 500 generations. The line represents the mean result of 100 replicated simulations, each spanning 500 generations. Pleiotropy on all traits means that pleiotropy affected both the quantitative trait under directional selection and unreduced gamete production. **A** No environmental factor; **B** environmental factor 0.1; **C** environmental factor 0.3.

At the beginning of the directional selection phase (step 2 of the model), the frequency of unreduced gametes, due to genetic effects, increased in the first generations and then decreased back to an equilibrium value (Figs. 4B and 5). This steep initial increase was due to the fact that, at the beginning of the adaptation process, selection favored the increased formation of newly adapted tetraploid individuals. Once diploid individuals were closer to the new phenotypic optimum, formation of tetraploids was either selected against and tetraploids remained in the minority within the population due to minority cytotype exclusion (between 0 to 10% of tetraploids when no environmental effect was modeled; Fig. 5A; between 0 to 55% of tetraploids with an environmental effect of 0.1 on UG production; Fig. 6B), or tetraploid formation was continuously favored until fixation of tetraploidy (100% of tetraploids when the effect of the environment on UG production was strong, independent of the mutation rate; Fig. 6C). As previously described, the overall effect of increased mutation rates was to increase both the average genetic production of unreduced gametes and of tetraploids within populations (Figs. 5 and 6). It was, however, notable that the mutation rate has a synergistic effect with the environmental effect on unreduced gametes: the higher the environmental effect, the higher the genetic response to increase UG production (Fig. 5). This is because UG production is under frequency dependence: the higher UG production is in the population, the less it is counter-selected. Thus, the stronger the effect of the environment, the easier it is for a mutation increasing genetic UG production to be maintained in the population. Pleiotropy had a generally negative effect by decreasing the frequency of unreduced gametes and tetraploids (Figs. 5 and 6). Interestingly, the effect of pleiotropy on limiting both genetic UG production and the frequency of tetraploids was stronger when pleiotropy was limited to UG production of both male and female functions, rather than affecting both genetic UG production and the quantitative trait under directional selection (Figs. 5B and 6B). This was due to the fact that if the mutation affecting both genetic UG production and the phenotypic trait had a positive effect on fitness (i.e. the mutation brought the phenotype closer to the new optimum), the mutation was selected despite its costly effect on UG production.

**Fig. 7:**
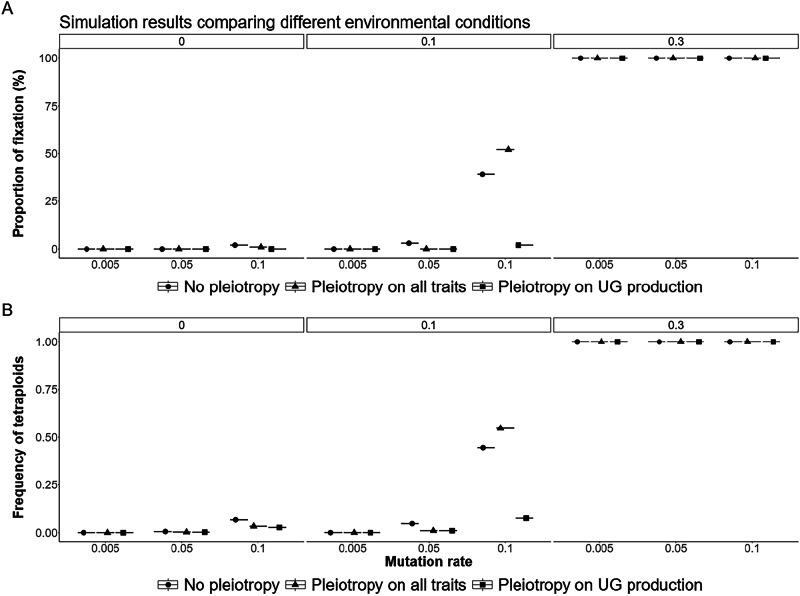
General effects of pleiotropy and environmental change on the evolution and probability of fixation of polyploidy. Effect of pleiotropy scenario (none, all traits, unreduced gametes; number of pleiotropic loci = 40) and environmental factor (0, 0.1, 0.3) on the **A** fixation and **B** frequency of tetraploids in the population under different mutation rates (*U* = 0.005, 0.05, 0.1; differently colored lines) for 500 generations. Pleiotropy on all traits means that pleiotropy affected both the quantitative trait under directional selection and unreduced gamete production.

Overall, the fixation of polyploidy remained rare (Figs. 6 and 7). With low mutation rates, populations adapted to new conditions either by remaining diploid, with the individuals adapting to the new conditions, or by forming mixed-ploidy populations of adapted diploids and tetraploids, notably when the effect of the environment on UG formation was high (Figs. 6 and 7). With higher mutation rates, the formation of a mixed-ploidy population was much more frequent, with tetraploids reaching 10 to 55% in the populations (Fig. 6B). The fixation of polyploidy was frequent when unreduced gametes swamped the population, i.e. with a high mutation rate and a strong effect of the environment (Figs. 6C and 7).

## Discussion and conclusion

We simulated the consecutive consequences of stabilizing selection and directional selection in response to an environmental factor during the origin and maintenance of autopolyploids. In this process, the pleiotropic effect of mutations on UG production and a quantitative trait affecting population fitness were tested. Under stabilizing selection, pleiotropy had a negative effect on the production and genetic variation of unreduced gametes. Under a realistic low mutation rate in the focal genes, we found no fixation of tetraploidy. Higher mutation rates led to higher heritable rates of UG production and to the formation of stable mixed-ploidy populations; however, with tetraploids remaining rare ( < 10% in frequency). Under directional selection, the environmental factor had a complex effect on UG production and population cytotype structure. Taken as an isolated parameter, the higher the effect of the environment on UG production, the higher the frequency of polyploidy at the end of the 500 simulated generations following the environmental change. In addition, environment and genetic architecture had a synergistic effect: the stronger the effect of the environment, the higher the genetic component of UG production. This is mainly because UG production is frequency-dependent, i.e. the higher it is, the less costly it is.

### Evolution of tetraploidy during stressful conditions

Environmental stress is considered to be important to the origin of polyploids, as it affects UG production (Kreiner et al. 2017a; Mason & Pires 2015). In particular, high and low temperatures have been shown to impact male UG formation (De Storme et al. 2012; Mason et al. 2011; Pecrix et al. 2011; Schindfessel et al. 2023; Wang et al. 2017), although the plants used therein were mainly of hybrid origin, which makes them generally more prone to form unreduced gametes. Treatment with high temperatures is commonly used to generate unreduced male gametes for polyploid breeding (Mai et al. 2019). In our simulations, tetraploids persisted in a population either under extreme environmental effects or through higher genetic mutation rates that increase UG production to ca. 20 to 40% at equilibrium (*U* = 0.05, 0.1; Fig. 6C; Supplementary Fig. 4). These results are in line with the recent Gerstner et al. (2024) model, which found that environmental stochasticity for UG production can lead to the evolution of polyploidy. However, as UG production in natural populations of non-hybrid species is typically much lower, ranging from 0.1 to 2% (Kreiner et al. 2017), our high mutation rates seem very unrealistic. If we assumed a realistic mutation rate for an average of 2% of UG production, tetraploids only got fixed in a population under a strong environmental effect in our simulations, while a moderate environmental effect resulted in a mixed-ploidy population with rare tetraploids ( < 10%; Fig. 6C). However, we found that the higher the effect of the environment on UG production, the higher the genetic increase of UG formation. If the environmental stress also affects the mutation rate (like U.V. exposure that could affect both temperature and alter the mutation rate; Gengyo-Ando & Mitani 2000), it is possible that higher rates of polyploid formation could be found.

In the first simulated generations after the onset of the directional selection phase, which represents the environmental change, we observed increased UG production and an increased frequency of newly adapted tetraploid individuals in the population compared to later generations (Fig. 5). Under directional selection, mutations leading to UG formation might be beneficial for tetraploid establishment, while counteracting diploid adaptation, and polyploids may initially benefit from phenotypic shifts associated with WGD. Phenotypic shifts associated with WGD were comprehensively summarized by Clo & Kolář (2021) and Porturas et al. (2019), and more generally reviewed by Bomblies (2020), all of them showing that synthetic neo-polyploids can be associated with selective advantages under some conditions, but not uniformly among the polyploid lineages studied.

Our findings are in contrast to those of Clo (2022b, partly due to differences in the hypotheses assumed in the models) in that the author found tetraploids to be associated with an initial decrease in their adaptive potential compared to their diploid progenitors, while within less than 100 generations of the simulation, they reached or exceeded the additive variance of the diploids. In our simulations, tetraploids were directly adapted and selected for in an initial period of around 50 generations, but then were selected against once diploid individuals were closer to the new phenotypic optimum. They then remained in the minority within populations, resulting in mixed-ploidy populations under moderate environmental effects. Even if the link between polyploidy and adaptive potential is probably highly multifactorial (Clo 2022a), modeling non-adapted tetraploids with smaller genetic diversity might have favored the diploid cytotype in mixed-ploidy populations.

Especially for plants, mixed-ploidy populations have been frequently reported in multiple diploid-autotetraploid species, with varying proportions of diploids and their polyploid derivatives (Kolář et al. 2017). The strength of selection against polyploid cytotypes in these populations further depends on several factors. Gene flow between ploidy levels may transfer novel adaptive alleles (e.g., in *Arabidopsis arenosa*, Baduel et al. 2018; Australian burrowing frogs of the genus *Neobatrachus*, Novikova et al. 2020) and may contribute to tetraploid persistence in the long term, but we did not model this scenario, as we assumed tetraploids were directly adapted to the new environmental conditions. In our simulations, only under a strong environmental effect did tetraploids come to fixation. This is in line with the theoretical framework of Oswald & Nuismer (2011), who showed that tetraploid populations can adapt more efficiently than their diploid progenitors if the populations undergo an environmental change, and that this change is aligned with the phenotypic modifications found in tetraploid individuals. Both models also support the hypothesis that polyploids are expected to face higher extinction rates than their diploid progenitors in the absence of environmental change (Levin 2019). In contrast, polyploids may thrive in stressful environments and broaden their ranges, leading to their prevalence in such environments and during historical periods of climate change (Bomblies 2020; Rice et al. 2019. In sum, while our model did not include post-WGD environmental adaptation, it shows that environmental change may support polyploid establishment by directly affecting the rate of UG formation, moreover, with a further positive feedback effect on its underlying genes.

### The consequences of UG genetic architecture on polyploidy evolution

UG production is both environmentally and genetically determined (Parrott & Smith 1986; Tavoletti et al. 1991b). It varies among individuals within and between populations, such that most individuals in natural populations of non-hybrid species produce male unreduced gametes at zero or low frequency and only a small number of individuals produce substantially more (Kreiner et al. 2017a). UG mutations affect various cytological stages (Bretagnolle & Thompson 1995; Brownfield & Kohler 2011; De Storme & Geelen 2013), and they involve pre-meiotic, meiotic, and post-meiotic pathways. For example, in *Arabidopsis thaliana*, key proteins comprise DYAD, AtPS1, JAS, TES, MPK4 (meiotic), and INCENP, RBR (post-meiotic) (Brownfield & Kohler 2011). Genes involved in meiotic abnormalities and UG production have been found in other species (*Solanum sp., Medicago sp., Trifolium pratense, Petunia sp*.), as reviewed by Bretagnolle & Thompson (1995). This polygenicity may account for the variation in UG frequency in natural populations and the variance of UG production found in our simulations. A clear theoretical and empirical framework for the effect of genetic architecture on UG production, particularly pleiotropic effects, on the maintenance of polyploidy is lacking. It is unclear whether a few major-effect mutations or numerous small-effect mutations are involved, and how they affect the further trajectory of polyploid adaptive evolution (Bomblies and Peichel, 2022).

In our simulations, one of the main findings is that pleiotropy always has a negative effect on UG production and its variance. For example, a higher number of pleiotropic loci significantly reduces UG production (Fig. 4). This indicates that pleiotropy constrains UG production by increasing the cost of complexity, a general effect of pleiotropy (Bomblies & Peichel 2022; Orr 2000). While UG production and variance under pleiotropic effects are lowered during stabilizing selection in our simulations, the overall fitness of the population is often increased and thus seems to benefit from pleiotropic effects (Fig. 2). However, we found that during directional selection, if there is pleiotropy between UG formation and fitness-related loci, a mutation having both a positive effect for diploid adaptation and increasing UG production can be temporarily selected. The complexity of pleiotropy on fitness (positive effect) and UG production (negative effect) is in line with what is found with broader patterns of adaptation in nature: pleiotropy favors local adaptation at the short spatial scale (Whiting et al. 2024), but reduces global adaptation (Nocchi et al. 2024). It is known that mutations affecting UG formation in *Arabidopsis thaliana* also impact the quantity of pollen and/or ovules of individuals (Brownfield & Kohler 2011). If this kind of pleiotropic mutation can increase in frequency during an environmental change, as explored by us, polyploidy could evolve by chance at least at low frequency, highlighting once again the role of stochasticity in polyploid evolution (Clo et al. 2022; Gerstner et al. 2024; Kauai et al. 2023).

According to empirical data, UG production is a deleterious phenotype, maintained at a low frequency in the population (Kreiner et al. 2017b). The mechanisms for the maintenance of unreduced gametes remain unknown, but could involve mutation-selection balance (Kreiner et al. 2017b). In general, pleiotropy on quantitative traits can reduce the selection efficacy in natural populations (Fraïsse et al. 2019; Zhang 2023). Consequently, in our simulations, individuals with UG production faced strong purifying selection but were still maintained in the population. Environment and genetic architecture had a synergistic effect in our simulations: with both an environmental effect and pleiotropy, population fitness decreased. Reduction of population fitness resulting from pleiotropy could eventually lead to a reduction of the population size, and the population could then be more affected by drift. This scenario would be in accordance with previous models showing that the maintenance/fixation of polyploids is more likely when selective forces are relatively weak (Clo et al. 2022), favoring polyploids under environmental change.

### Limitations of the model

For our model to be informative, we had to avoid some genetic and ecological aspects related to ploidy for the model to remain understandable. Some alternative models exploring overlapping generations (van Drunen and Friedman, 2022) or stochastic effects of the environment (Gerstner et al. 2024) can be found elsewhere, so these hypotheses will not be discussed here. A first simplification is that we hypothesized triploid and higher polyploid levels to be non-viable. Triploids may theoretically play a crucial role in mixed-ploidy population dynamics (Husband 2004; Kauai et al. 2024). However, triploids are typically in the minority in natural populations of autopolyploids, possibly being abundant only during the earliest stage of polyploid formation in a population (Burton & Husband 2000a; Husband & Sabara 2004; Kolář et al. 2017). Also, we decided to model the fact that polyploidy is adaptive in the short-term, which can be true in some conditions (Jiang et al. 2022; Maherali et al. 2009; Wang et al. 2019; Wu et al. 2019), but not in general (Clo & Kolář 2021; Porturas et al. 2019). Considering equivalent fitness for diploids and tetraploids or a decrease in fitness in polyploids would not have drastically changed the results. If the population is swamped with unreduced gametes (high mutation rate and strong environmental effect), tetraploids would fix in a population independently of their fitness (see Clo et al. 2022 for an example). However, for most simulations for which we found mixed-ploidy populations it is likely that with no tetraploid fitness advantage, the populations would have remained diploid most of the time.

We modeled obligately outcrossing populations, while any form of assortative mating (i.e. avoiding crossing among cytotypes such as pollinator preferences or strong self-fertilisation) or spatial structure favoring mating within cytotypes could increase the likelihood of fixation of polyploidy (Otto & Whitton 2000). The main reason is that the aim of our model was to simulate a potentially realistic architecture of UG production, and that assortative mating should not modify the outcomes of previous models, as the advantage related to assortative mating is independent of the architecture of UG production. Finally, we did not model demographic events following the environmental change. One of our major results is that for realistic simulations (with UG production fitting empirical data) the adaptation of populations to new conditions is more likely by diploid individuals reaching the new phenotypic optimum rather than by adapted tetraploids reaching fixation. We could have simulated fluctuating population size, with low-fitness individuals (diploids in our case) going extinct before reaching the reproductive stage, for example. With a lower population size, any increase in tetraploid frequency would have lowered the strength of frequency-dependent selection more efficiently than in our simulations, possibly leading to mixed-ploidy or tetraploid populations more often.

## Supplementary information

Supplementary figures 1 to 4

## Data Availability

Simulation code is available at https://github.com/JosselinCLO/Model_UG_pleiotropy_environment.
