# Supplementary material for: How environment and genetic architecture of unreduced gametes shape the establishment of autopolyploids
Source: Heredity (Edinb). 2026 Jan 13;135(2):55–66. doi: 10.1038/s41437-025-00816-3 (PMC12891648; doi:10.1038/s41437-025-00816-3)
Supplement: Supplementary file 1 — Supplementary figures 1 to 4 [file 41437_2025_816_MOESM1_ESM.docx]

Supplementary data


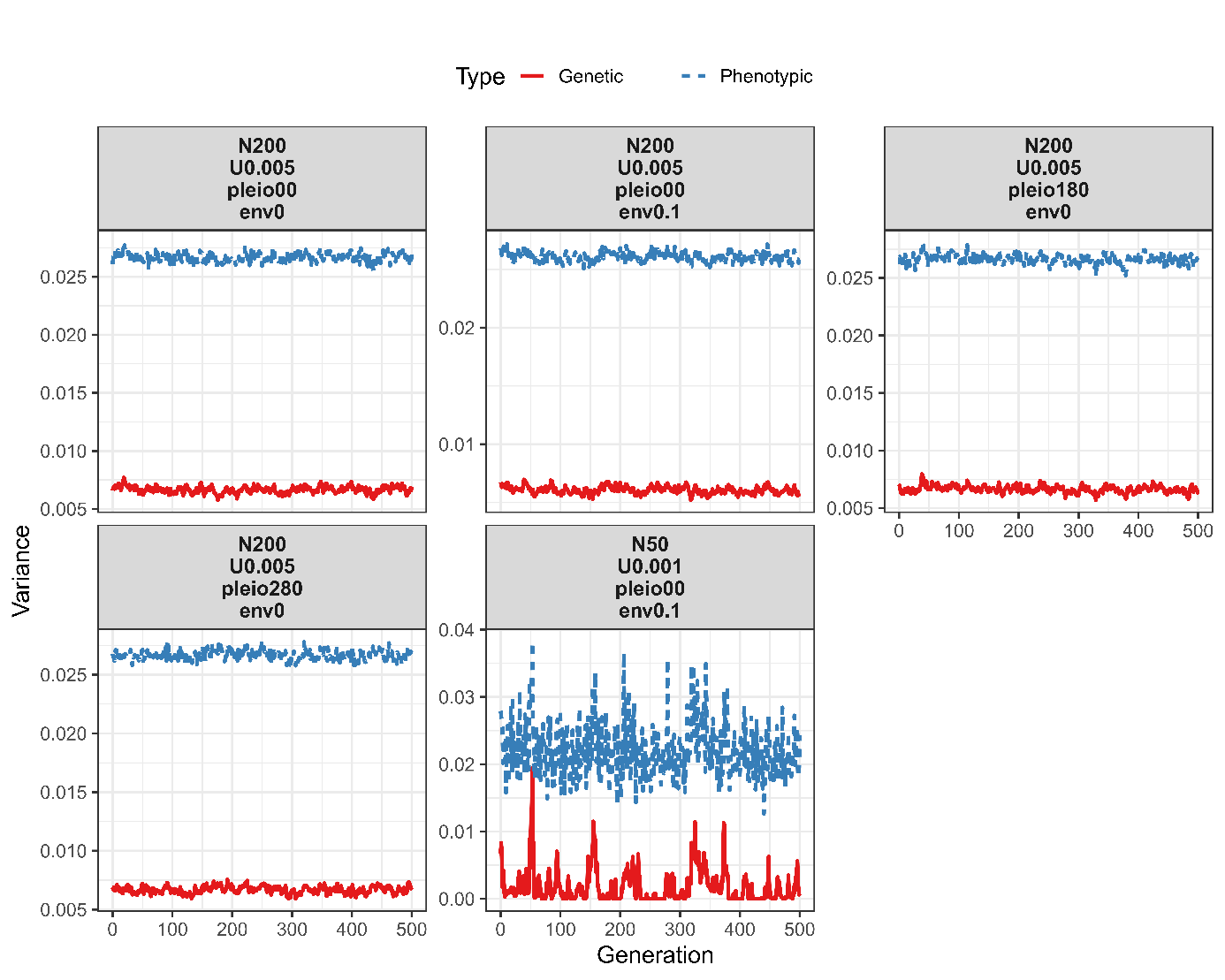


**Figure S1.** Comparison between genetic and phenotypic variance during the directional selection phase, for the realistic parameter set (see main text for details). N stands for population size, U stands for the genomic haploid mutation rate, and env stands for the environmental effect on unreduced gamete production (+0, +0.1). Pleio X00 stands for the level of pleiotropy (X=0 no pleiotropy, X=1 pleiotropy between female unreduced gamete production and fitness, X=2 pleiotropy between male and female unreduced gamete production and fitness). When pleiotropy is modeled, 80 loci are pleiotropically linked.


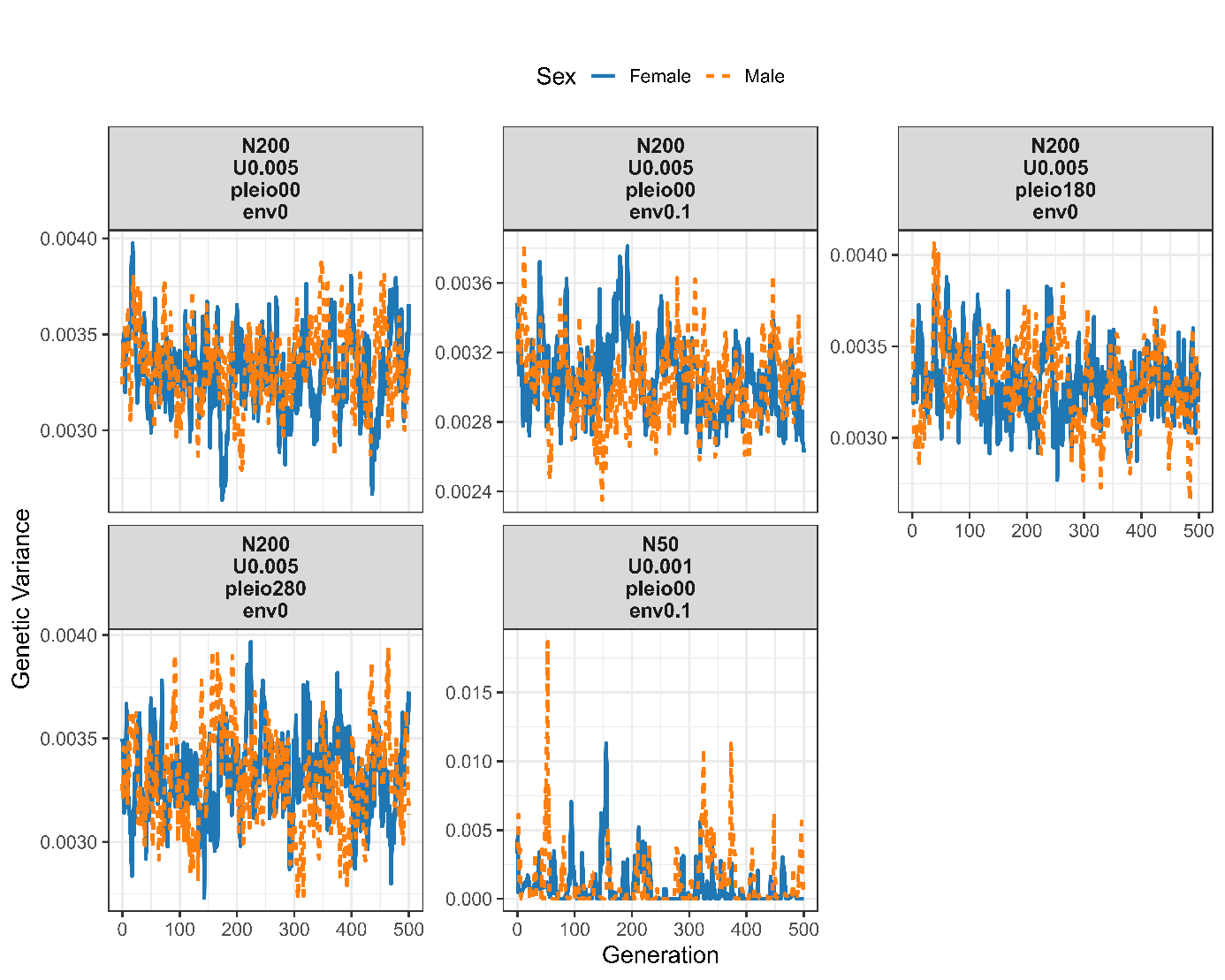


**Figure S2.** Production of male and female unreduced gametes for the realistic parameter set (see main text for details). N stands for population size, U stands for the genomic haploid mutation rate, and env stands for the environmental effect on unreduced gamete production (+0, +0.1). Pleio X00 stands for the level of pleiotropy (X=0 no pleiotropy, X=1 pleiotropy between female unreduced gamete production and fitness, X=2 pleiotropy between male and female unreduced gamete productions and fitness). When pleiotropy is modeled, 80 loci are pleiotropically linked.


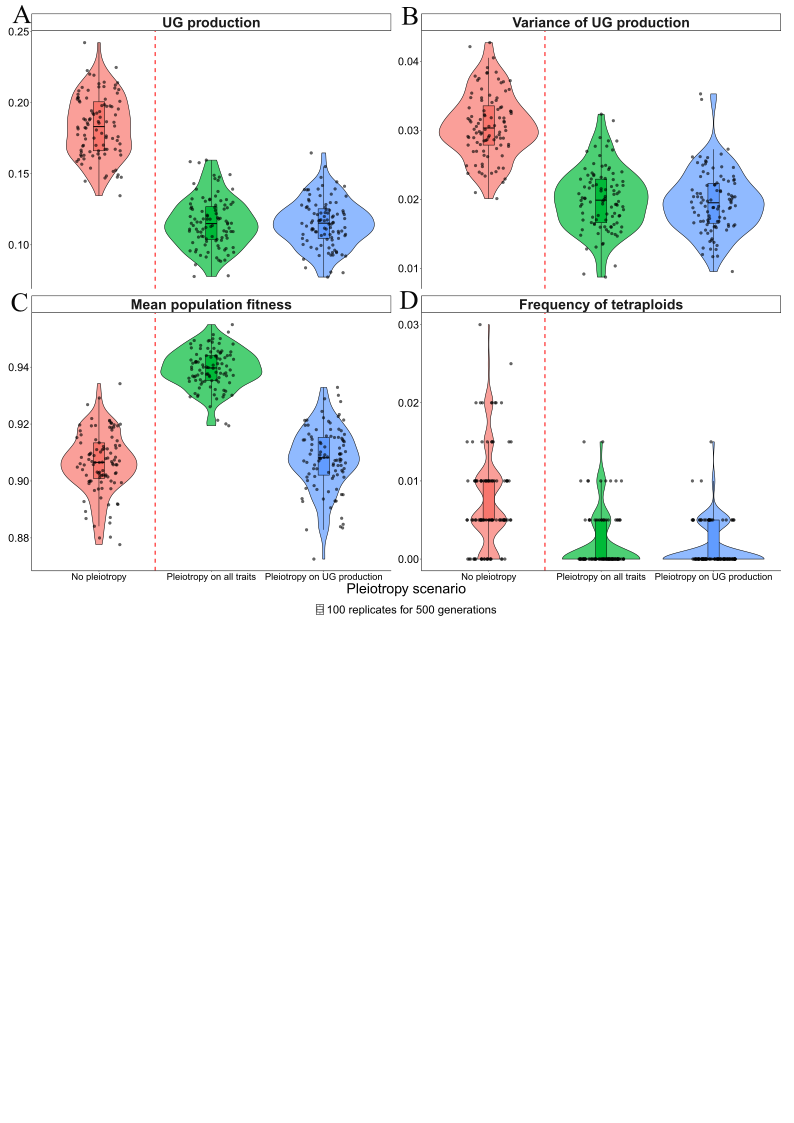


*Figure S3: Effect of pleiotropy scenario (no, all traits, unreduced gametes; number of pleiotropic loci = 80) on unreduced gamete production and its genetic variance, population fitness, and tetraploid frequency at the mutation-selection-drift equilibrium, simulated from a population of genetically identical diploid individuals under the mutation rate U = 0.05. Simulations were run for 100 replicates; black dots represent individual replicates. Pleiotropy on all traits means that pleiotropy affected both the quantitative trait under directional selection and unreduced gamete production. (A) Proportion of unreduced gamete production in diploid individuals; (B) variance of unreduced gamete production in diploid individuals; (C) population fitness; (D) frequency of tetraploids in the population.*


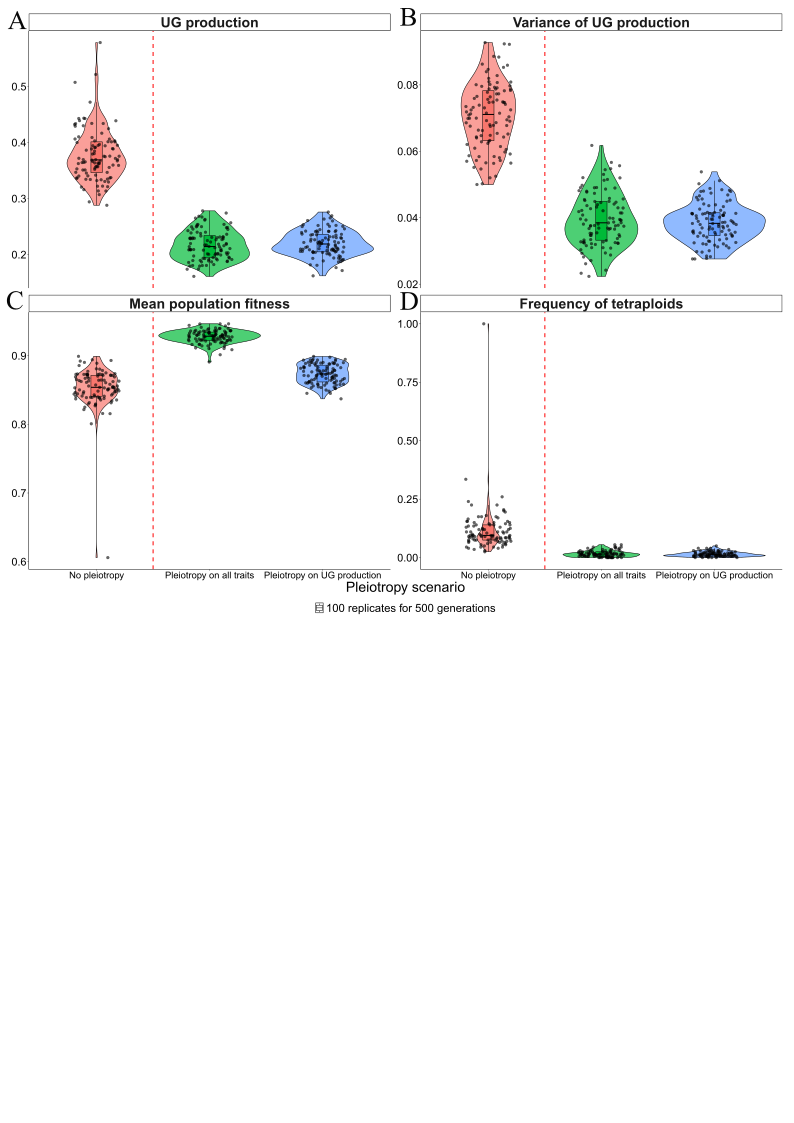


*Figure S4: Effect of pleiotropy scenario (no, all traits, unreduced gametes; number of pleiotropic loci = 80) on unreduced gamete production and its genetic variance, population fitness, and tetraploid frequency at the mutation-selection-drift equilibrium, simulated from a population of genetically identical diploid individuals under the mutation rate U = 0.1. Simulations were run for 100 replicates; black dots represent individual replicates. Pleiotropy on all traits means that pleiotropy affected both the quantitative trait under directional selection and unreduced gamete production. (A) Proportion of unreduced gamete production in diploid individuals; (B) variance of unreduced gamete production in diploid individuals; (C) population fitness; (D) frequency of tetraploids in the population.*
